# The Limits and Possibilities of Cause of Death Categorisation for Understanding Late Nineteenth Century Mortality

**DOI:** 10.1093/shm/hkac040

**Published:** 2022-08-30

**Authors:** Angélique Janssens, Isabelle Devos

**Affiliations:** Department of History, Radboud University, Nijmegen, The Netherlands; Faculty of Arts and Social Sciences, Maastricht University, Maastricht, The Netherlands; Department of History, Ghent University, Ghent, Belgium

Over the last decade, the number of large-scale databases in research on the great mortality decline and the epidemiological transition that has taken place since the eighteenth century has increased enormously. Moreover, the advances in Information and Communication Technology (ICT) and quantitative methods have greatly enriched the uses that can be made of such datasets. Yet, comparative research is severely hampered because diagnostics and nosology have varied over time and across countries, regions, localities and even among doctors. As a result, historical cause of death registration and classifications can be problematic in many respects and require careful interpretation. The introduction of this special issue centres on the challenges posed, but also the opportunities offered, by the use of historical cause of death statistics in mortality research.

In this introduction, we first discuss the academic debate in which most historians using cause of death data situate themselves, and why these types of data can help illuminate the substantive issues of that particular field. Following that, we present a brief overview of the different cause of death classification systems that researchers have used to analyse the nineteenth-century cause of death data focussing on the pitfalls and possibilities of each system. This is followed by a discussion of the four articles in this special issue. Some authors make use of ICD-10 (the 10th revision of the *International Classification of Diseases*), others combine different systems or construct a new one specific to their historical source or research aims. We compare and critically assess their use to examine historical changes in mortality and cause of death patterns. Finally, we also make a plea to develop interdisciplinary efforts in this field. The long-run changes in mortality have so far been the almost exclusive domain of historical demographers. The field would benefit from strong interdisciplinary cooperation with medical historians and epidemiologists.

## Background: Long-Run Changes in Mortality and Epidemiology

Long-run changes in mortality and life expectancy count among the great revolutionary changes of the past 200 years.^1^ Substantial reduction in mortality caused life expectancy to increase from an average of as low as 30–40 years in the early to mid-nineteenth century to as much as 84 years in some countries of the western world today. In tandem with these mortality changes, we find radical changes in epidemiological profiles. The type of diseases people are suffering from today, and the causes of death people die of, have changed in important ways. In the western world, infectious diseases have given way to cardiovascular diseases and various types of cancers, the so-called epidemiological transition.^2^ Causal factors threatening our health today have changed accordingly. Whereas in the past, mortality risks were greatest for infants and young children, today, the burden of disease and mortality is coming down primarily in the highest age categories. In other words, the patients, doctors are seeing today, are vastly different from the ones who were calling on their help in the nineteenth century.

These entangled and interrelated changes in mortality and disease are still not fully understood. A large part of these transformations took place long before the arrival of modern-day curative medicine, such as antibiotics and mass vaccination programmes, which in most western countries only appeared in the 1940s and 1950s. Debates are continuing as to what caused these improvements in life expectancy before the 1940s, whether it is increases in living standards and hence nutritional intakes, improvements in public and/or personal hygiene, behavioural change, infant feeding practices or higher levels of education for certain groups within the population. This is a complex puzzle to resolve, which requires multi-disciplinary approaches spanning the fields of history and historical demography, medical history and historical epidemiology. It also requires innovative data and data technologies, ideally incorporating cause of death data at the individual level. Cause of death data at the individual level will greatly enhance our understanding of mortality patterns and changes over time. Knowing what disease someone died of, will crucially elucidate our understanding of why that person died in that particular place, at that particular moment in time and at that particular age. It will enhance our capacity to better capture what American medical historian Günther Risse once called the ‘shifting ecology of disease’, the complex and dynamic interactions between biological and non-biological factors impacted upon changing disease patterns.^3^ Aggregated cause of death statistics, by comparison, pose the risk of revealing only spurious trends in shifting patterns of death and disease. This problem is exacerbated by the complex interrelated changes occurring in medical science, in social and economic contexts and changing bureaucratic procedures, by state or other authorities, surrounding the drafting of causes of death. However, we wish to underline that these aggregated statistics should not be ignored by historians. In fact, in this special issue, one of the papers, by Nynke Van den Boomen, demonstrates ways how to test and probe these types of aggregated data to make them fruitfully speak to us on the nineteenth-century mortality decline.

It should be clear, however, that historical cause of death data is not unproblematic, whether they come in the form of individual-level data, without being obscured by historical nosologies, or in the aggregated format. They can never be used in historical research without extensive internal data checking through complex routines assessing patterns, deviations and anomalies in the use of disease terms, for example, over time and/or location, by age, gender, disease categories, etc. The ICT and data revolutions also make this an easier job to execute, especially when datasets are available for millions of individuals over long periods.^4^ The challenges involved in the use of historical cause of death data and classification systems have already been discussed before by historical demographers and medical historians.^5^ Since then, the data revolutions taking place in many countries, with digital techniques making large numbers of a great variety of source material easily available to researchers, have expanded the possibilities for careful and critical use of cause of death data. Cause of death data can now be compared with contemporary medical reports and medical knowledge, as well as information on how registration practices were organised.

## On the Registration and Classification Systems for Cause of Death Descriptions

Attempts to identify and classify individual diseases and causes of death go back a long way in history.^6^ Although some groupings of diseases were already apparent in the humoral theory of the ancient Greeks, the first classification of diseases is generally credited to the French physician François Bossier de Lacroix, better known as Sauvages, who published in 1763 in his *Nosologia Medica* a system listing 10 major classes of diseases and 2,400 individual diseases. Still, the systematic involvement of physicians in these attempts has emerged only very recently, that is, from the late nineteenth century onwards. Before that time, mainly state and city authorities were involved, and in some countries, most notably the Scandinavian countries, from the eighteenth century onwards religious authorities were the main actors and bodies driving and determining registration practices of diseases and causes of death.

Religious authorities had already begun to record causes of death systematically in the seventeenth century. This practice became even more widespread in the eighteenth century. Local parishes in many European countries kept registers of vital events (births, marriages and deaths) to monitor their populations and the degree of religiosity of their flock. In particular, in the Nordic countries, these registers were well kept and provided a wealth of information. For instance, in Norway, from the early decades of the nineteenth century onwards, the priests were also obliged to register the cause a person had died of. To fulfil their duties adequately, the priests also received some medical training, and by the mid-nineteenth century, it appears that the parishes had managed to set up a complete recording of causes of death in the burial registers. However, the variation between parishes and priests was likely quite large, so comparability between regions and communities within the country was nearly impossible. Hence, Norwegian national authorities began to impose regulations on doctors and priests as well as an official nomenclature of diseases in 1861.^7^ That list provided 122 standardised causes of death and included instructions on how to use the Norwegian and Latin terms. This development was initiated and inspired by the first Statistical Congresses taking place in 1853 and 1855, in Brussels and Paris, respectively.

Similar developments were taking place in other western countries. The development of a cause of death registration occurred in most cases against the background of a growing governmental interest in collecting statistical data on all kinds of demographic, economic and social processes. Medical professionals and hygienists, in particular, stressed the need for objective measures of health, including empirical research, standardisation of data collection and increased dissemination of data. In Belgium, for instance, a national initiative for the compulsory cause of death registration was taken in 1851 in response to a series of epidemics in the 1840s. Besides the cause of death, specific data had to be collected using pre-printed forms with the name of the deceased, surname, sex, occupation, age, marital status, and date and place of death. Although the cause of death had to be determined by a doctor, it was common practice for civil servants to fill out the certificates, usually relying on family members or neighbours to provide the cause. In large cities, however, a doctor was appointed for the task.^8^ Also, in Sweden and the Netherlands, the central state began to issue regulations requiring that the cause of death be recorded by a physician. In Sweden, that was the case in 1860, whereas the Netherlands followed a few years later in 1869 as a result of various local initiatives by physicians.^9^ This obligation did not immediately receive a warm welcome from all Dutch physicians, some of whom regarded this as a breach of patient confidentiality. In 1875, the Dutch Ministry of the Interior introduced a national classification system consisting of 34 categories, with 33 of them consisting of disease categories and the 34th counting the numbers of deaths that had received no medical treatment. In Belgium, three doctors, members of the Belgian Central Commission for Statistics, drew up a list of 116 causes of death which served as the first Belgian nomenclature in 1867. These national classification systems were a mix of vague terms denoting symptoms, such as intermittent fever, weakness or convulsions, and other more precise terms for ailments that could be diagnosed more easily, such as smallpox or measles.

While initiatives were taking place at the national level, the growing internationalisation in the area of medical statistics also had an impact. In 1900, at an international conference in Paris, efforts to arrive at an international classification standard to make the cause of death recording comparable across all countries were finally successful and resulted in the ‘International Classification of Diseases’. The first ICD consisted of 179 diseases and an abridged classification of 14 groups. It was a slightly revised version of the Jacques Bertillon classification of causes of death used by the city of Paris which was a synthesis of English, German and Swiss classifications.^10^ The classification was largely based on William Farr’s principle of distinguishing between general diseases and those localised to a particular organ.^11^ ICD-1 was translated from French into several other languages and its use spread rapidly around the globe. Since then, the ICD has been revised almost decennially, taken account improved medical knowledge and new diagnostic techniques. Currently, it is maintained by the World Health Organisation and is the most widely used statistical classification system for diseases in the world. The latest revision, the ICD-11, came into effect on 1 January 2022.^12^ With around 55,000 codes, it is almost five times as big as the ICD-10. The lack of coherence in the successive revisions of ICD has strongly restricted its application in historical research.

To facilitate causes-of-death studies across time and place, several solutions have been proposed. According to Alter and Carmichael, one option is to apply a broad grouping of diseases to guarantee as best as possible that each cause of death is placed under the same heading from one period to another. Other possibilities include focussing on specific causes of death that were consistent over time or limiting the analysis to a period with a homogenous nomenclature. More ambitiously, some scholars have developed their own classification method. French demographers Jacques Vallin and France Meslé, for instance, constructed in the late 1980s a system for aggregated data by providing bridging codes and correspondence tables for the ICD revisions. Enabling the transfer of causes of death from one revision to another, their method has made it possible to create nosologically continuous causes of death data for countries such as France and the Netherlands across the twentieth century.^13^ Researchers remain nonetheless limited by other constraints of the ICD since the earlier revisions lack the detail needed to accurately reflect the medical terminology and procedures of the time. In the 1990s, historian Joseph Bernabeu established a method for individual-level cause of death data, which he further developed in 2003 with other Spanish researchers.^14^ Their system, the BeRaSaRo-system, is based on a double classification: the one proposed by Bertillon using anatomical criteria and another by Thomas McKeown based on disease aetiology.^15^ As shown in different Spanish case studies, the method distinguishes causes of death according to their mode of transmission (air-, water- and food-borne diseases, and other means) and therefore provides better insight into the key determinants of death. Whilst the double classification is very flexible, the method loses much of its use for research of the advanced stages of the epidemiological transition when non-infectious diseases dominate the mortality pattern.

Individual-level cause of death data are also key to the European network called SHiP+ which brings together historical demographers, medical historians and historical epidemiologists in the most recent attempt to study health changes across time and space.^16^ SHiP+ began in 2017 as a network focussing on port cities only, hence the acronym SHiP (Studying the history of Health In Port cities). Recently, the network has decided to expand its activities to include non-port communities in Europe. This has increased the number of participating scholars and the number of regions and countries within Europe that can be studied. One of the main aims of the network is to construct an international historical coding system for disease terms, called ICD-10h, which can be used across countries and languages, and for longitudinal approaches connecting the eighteenth century to the present day.^17^ The connection with modern disease patterns is possible as the coding system is an adaptation of the ICD-10. Currently, the prototype has been produced and is now being tested on a set of studies in eight different European countries.^18^ The network will also produce a set of classifications to be used for different age groups and research purposes. The coding and classification systems will ultimately be made available as open access tools to the entire research community. These tools will facilitate truly comparative work between European countries over long periods, and will therefore permit researchers to make important contributions to the study of long-run changes in mortality and epidemiology.

## The Articles in This Special Issue

The four articles in this special issue make use of cause of death data which are taken from different types of sources (parish registers, hospital records, death certificates, cause of death registers) as well as from different countries in Europe. All scholars make use of large-scale datasets, one of them (the Netherlands) even encompassing an entire country, the others large- or medium-sized cities (Copenhagen, Venice, Trondheim, Roosendaal). Each of these papers is focussing on crucial changes in the history of mortality and survival, ranging from infant mortality to adult mortality, and from water- and food-borne diseases, to tuberculosis and maternal mortality. The paper on the rich Venetian cause of death data furthermore shows how these data can contribute important insights to more traditional topics in the field of medical history, namely the history of hospitals and how these institutions developed over time.

The first article in this issue by Nynke van den Boomen provides a nice showcase of how to test a nineteenth-century cause of death classification before using the data in historical research. The author aims to uncover the strengths and weaknesses of the first national classification system used to register all causes of death in the Netherlands occurring between 1875 and 1899. This register consists of a series of aggregated statistics which compiles all mortality data by municipality into 5-yearly overviews, distinguishing 34 disease categories and a separate category for deaths without medical treatment. The author asks whether and how this source material might be treated in a useful manner in historical demographic and epidemiological research for the study of infant mortality. This is done through a meticulous comparison to detect anomalies and deviations between periods and provinces. In the case of the Netherlands, this is a good choice as the health inspectorate was also organised at the provincial level, and these inspectorates may likely have influenced the registration of causes of death as well as the tabulation of diseases within their region of authority. She modifies the nineteenth-century nomenclature to make it suitable for the study of infant mortality. This sensitivity to age is an important warning to other scholars who wish to study any historical or contemporary nosology. The results of the extensive testing conducted by Van den Boomen show that pitfalls in the material are in particular related to old medical terms such as debility and consumption, diarrhoea, acute diseases of the digestive system, arrested development, convulsions and trismus and epilepsy. She concludes that there is ample proof that medical practitioners and the clerks constructing the aggregated tables, intentionally as well as unintentionally misdiagnosed and miscoded the causes of death, and that in this period diagnostic and coding procedures were dynamic processes. This conclusion does not lead her to renounce the use of these types of statistics for historical research. However, she rightly issues a warning and urges scholars to seriously take into account the institutional and social–cultural history of these types of material before using it in the study of mortality.

Whereas the previous article was focussed on a historical classification system designed in the second half of the nineteenth century, the article by Barabara Revuelta-Eugercios, Helene Castenbrandt and Anne Løkke is targeted at two different modern classifications: the ICD-10 and the BeRaSaRo-system developed by Spanish researchers. This latter system follows closely the main distinctions as proposed by Thomas McKeown between infectious, non-infectious and ill-defined diseases. While acknowledging that historical causes of death are not meaningful as causes of death according to the standards of modern medical science, the authors very adequately set out how these data can be exploited. Their argumentation follows closely the line set out in the previous paper by Van den Boomen: only use historical cause of death data after a thorough analysis of the data and the analytical steps taken in the research project. Moreover, careful attention should be given to the specific time and place to which the research questions are related, as well as the medical thinking of that period.

The main body of this article is devoted to a test of the two classification systems on a dataset with the causes of death for circa 11,100 individuals that had died in the city of Copenhagen in the years 1880–1881. After careful standardisation of the cause of death expressions, there were 1,123 unique historical terms. What the test, not quite unsurprisingly, shows is that when applying an unadapted version of the ICD-10 this yields a high number of cases (16.6 per cent) in chapter 18, the chapter that is reserved for ill-defined causes of death. For infants, this number is even higher, 34.2 per cent, also quite according to expectations. The BeRaSaRo-system does a better job with only 4 per cent of ill-defined cases overall and 6 per cent for infants. The authors conclude that when dealing with large historical databases it is preferred to use a method based on multiple coding to incorporate the strengths of different systems. As part of that strategy, they also integrated the classification system designed by Bertillon in 1893. This clearly demonstrates the principle that a high degree of analytical sensitivity is required in research based on the historical cause of death data, and therefore makes a good guide for scholars new to the field. What this article also demonstrates is the great advantage of having individual-level cause of death data. These types of data generally have a higher degree of detail, compared to the aggregated statistics that Van den Boomen has to deal with (see above), and which enable the researcher to code individual diseases into various classification systems of his/her own choice.

The study by Renzo Derosas and Cristina Munno in this special issue is also based on individual-level cause of death data, this time taken from nineteenth-century death certificates for the large Italian city of Venice. The article aims to contribute to the history of European hospitals by examining where and why Venetians died around the middle of the nineteenth century. Were most deaths occurring at home or in one of the city’s hospitals, and what did people die of? Were some health conditions more frequently treated in hospital than at home? By contrasting hospital deaths with those occurring at home the authors intend to reveal the gradual modernisation of the hospital system in Venice, transforming from a place where people primarily came to die to a place where they were seeking to be healed. Their main target is to challenge Whiggish narratives that represent hospital history as an increasing progression towards full medicalisation. Venice is an excellent location for such a study given that it boasts a long tradition of care for the needy and the sick dating as far back as the tenth century. By the mid-nineteenth century, the Civic Hospital was a major medical institution consisting of 60 wards with a total of 1,200 beds, employing 10 head physicians, 20 surgeons, and 100 nurses. In addition to this large hospital, the city also counted a great number of smaller hospitals and hospices.

The death certificates that have been used for this study are of exceptional quality. Detailing a wealth of information, the death certificate had to be completed and signed by a doctor before a body could be buried. The doctor was also expected to go to the deceased’s residence to examine the body which enhances the quality of the cause of death of information on the certificate. To group the causes of death, the authors opt for a nineteenth-century classification system that was used in Venice at that time. This choice is typically motivated by research concerns. There is no need to connect to present-day epidemiological patterns or conceptions of diseases. Instead, they wish to connect to disease conceptions doctors were following at that time when deciding whether or not to treat patients in the hospital.

The authors examine all deaths occurring in the city in 1854 and 1869 differentiated according to where the death occurred, in a hospital or at home. By linking to the city’s wonderful population registers, the authors are able to analyse the impact of age, gender, socio-economic status and the presence of family members in the household on the likelihood of dying in hospital or at home. They find that those who died in hospital were mostly adult or elderly males, coming from the poorer classes, and with few relatives to support them. This confirms the hypothesis that mid-nineteenth-century hospitals were still a place for the poor, the destitute and the lonely. However, they do not regard this as a huge contrast with modern current-day hospitals. Even today, the ones who die in hospitals rather than at home are still the ones with small family networks and with fewer resources. However, based on an analysis of the causes of death, the authors also find that the doctors of the Venice Civic Hospital were at that time already pursuing some degree of medical specialisation. They appeared to have a special interest in diseases related to the cardio-circulatory system, and their admission policy did not make a difference between the poor and the rich. This results in the interesting conclusion advanced by the authors that to some extent the medicalisation of the hospital and the doctors was proceeding faster than the medicalisation of the patients.

Many medical historians may agree that causes of death descriptions are social constructions, not only in the past but also today. This idea that causes of death are generated within the context of discourses, rules and social norms predominant in society is precisely the starting point of the article by Hilde Sommerseth and Evelien Walhout. The focus here lies on how notions of gender affected disease conceptions in the past. Obviously, diseases may differ between men and women because of biological differences, which the authors relate to the biological category of sex. For instance, the biological differences are significant in early life: female babies are less likely to die of congenital diseases than male babies. Moreover, mortality hazards generally also favour adult women as they are less susceptible to infectious diseases due to genetic and immunological factors. However, differences in epidemiological patterns may also arise as a result of the social–cultural category of gender. Death and disease are gendered through cultural notions of masculinity and femininity, and through differences in socialisation, lifestyles, occupations, risk-taking behaviours, and other aspects of gender roles and positions. In the European past, above all in rural communities, these gendered patterns sometimes even contributed to excess female mortality, primarily for adolescent and young adult women.^19^

By using detailed individual-level cause of death data for two towns, Trondheim in Norway and Roosendaal in the Netherlands, the authors wish to explore how gendered notions of diseases framed the way mortality and cause of death were reported for adolescents and young adults. The way causes of death were reported in the past varied greatly between time and place. However, we have little evidence on whether that was also the case between men and women, although we do know that, for instance, diseases such as cancer, hysteria and melancholia were typically constructed as female. Whether in the face of death, the reporting and registration of the causes of death were also informed by sociocultural constructions of gender is unclear. The detailed individual-level cause of death data these researchers have at their disposal enable them to construct their own categories and single out specific diseases of interest. They opt to make use of an adaptation of the BeRaSaRo-system, developed by Spanish researchers and which we have already seen at work in this special issue, in the study on Copenhagen.

The data enable them to focus closely on sex differences in diseases that are most likely to have been affected by both biological and behavioural factors, namely airborne infectious diseases, and more specifically, TB and pneumonia. Their findings show an excess mortality for adult men over adult women in both the Norwegian and the Dutch communities. For both men and women in the adult age group, airborne diseases, most notably TB, were driving the mortality hazards, but the mechanisms behind these patterns were probably different for men and women. Industrial employment in unhealthy factories may have been the key factor for men, whilst married women may have been facing risks due to their domestic duties which kept them inside cramped and badly aired houses, often caring for sick children and other family members. The two communities were also risky places for men because of the higher risks of accidents and work-related injuries. The authors note that these latter hazards were removing men from the set of individuals who might have fallen victim to TB or other infectious diseases if they had not died earlier. In that sense, these different groups of causes were working as an interrelated set of urban hazards. For women competing risks were located in puerperal fever and breast cancer, which were both easily recognisable for doctors and priests registering deaths.

The great advantage of having individual-level cause of death data is also exemplified in this study. The authors conduct a detailed and thorough analysis of local registration practices in the two communities. To what extent were registration processes, and the nature of the causes of death equally detailed in the two communities is the question they ask. For instance, given the importance of TB in the overall mortality pattern of adult men and women, the authors take great pains to show the different but also equal ways in which TB was recorded. Finally, they convincingly show that the Trondheim priests who were doing the recording of the causes of death were very elaborate in the disease descriptions they were using, and were probably also at liberty to use a greater variety of disease terms than their physician colleagues in the Dutch city.

## Conclusions

While tackling issues central to research on the nineteenth-century mortality decline, the articles in this special issue have provided better and novel insights into the registration and classification of causes of death, be it either by doctors, priests or civil servants. The authors of the four articles are clearly devoted to the critical use of cause of death data, and show excellent examples of how researchers can make such data work. The studies on Copenhagen, Venice, and the comparative study on Norway and the Netherlands demonstrated that the analytical opportunities in mortality research can be greatly enhanced when the individual-level causes of death are available. The analytical power is increased, first of all, because individual disease terms can be identified without being hidden behind historical nosologies. The individual-level data enables researchers to construct their own detailed classifications. Although this limits the comparability of the studies, it allows the authors to test these classifications in various ways. Classifications should always be adequate in light of the research objective of the study. Hence, in the study on Venice, the authors opted for a contemporary nosology as they want to stay close to the medical thinking of Venetian doctors in the nineteenth century. For the other two studies, such a choice would be less adequate. The comparative study on Norway and the Netherlands, for instance, has a particular interest in infectious diseases; therefore, the classification they use is geared towards that goal. In this way, in-depth health changes can be studied. A second reason why these individual-level causes of death data greatly increase analytical opportunities is their richness. The causes of death registers contain a host of additional information, such as date of death, age, sex, marital status, address and occupation of the deceased. This produces a multitude of avenues for research into changing epidemiological profiles over time, as well as the social, economic and demographic dynamics behind these changes. Still, aggregated statistics should not be ignored by researchers. In fact, in this special issue, Van den Boomen demonstrated ways to test and probe these types of aggregated data to make them fruitfully speak to us on the nineteenth-century mortality decline.

The studies furthermore clearly show the importance and need of collaborating with medical historians. Understanding changes in medical thinking, as well as changes in the processes of diagnostics and the practices of registration, are essential for using historical records on causes of death. It is obvious that medical knowledge of diseases in the past was not as extensive or as adequate as it is today, and that aetiological thinking changed radically over the past 200 years or so. Moreover, improved diagnostic techniques and better recording practices can vary between geographic locations, as causes of death are shaped by their national, regional and local contexts. Therefore, it is important to know how the medical professionals and government officials perceived the diseases at the time and how this changed. As such, the registration of causes of death is closely intertwined with the social history of medicine.

## Footnotes

1 A large number of studies have been published on this exceptional development in the history of mankind. Some examples: Johan P. Mackenbach, *A History of Population Health: Rise and Fall of Disease in Europe* (Leiden: Brill, 2020); James C. Riley, *Rising Life Expectancy. A Global History* (Cambridge: Cambridge University Press, 2001); Roderick Floud *et al*., *The Changing Body. Health, Nutrition, and Human Development in the Western World Since 1700* (Cambridge: Cambridge University Press, 2011); Angus Deaton, *The Great Escape: Health, Wealth, and the Origins of Inequality* (Princeton, Princeton University Press, 2013).

2 Abdel R. Omran, ‘The Epidemiological Transition: A Theory of the Epidemiology of Population Change’, *Milbank Memorial Fund Quarterly*, 1971, 29, 509–38. For a recent overview of the epidemiological transition, see: Alexander Mercer, *Infections, Chronic Disease, and the Epidemiological Transition: A New Perspective* (Rochester: University of Rochester Press, 2014).

3 Günther B. Risse, ‘Cause of Death as a Historical Problem’, *Continuity and Change*, 1997, 12, 175–88.

4 Some of the largest data projects that have cause of death data are: Digitising Scotland, aimed at digitising data on 18 million individuals from Scotland back until 1856; Link-Lives, in Denmark which aims to reconstruct the lives of all Danes between 1787 and 1968; POPLINK at Umea University which will contain around 700,000 individuals in one of the northern regions of Sweden, as well as POPUM which covers four areas in the country with data from church records between the seventeenth century and approximately 1910.

5 George Alter and Ann Carmichael, ‘Studying Causes of Death in the Past’, *Historical Methods*, 1996, 29, 2, 44–48; George Alter and Ann Carmichael, ‘Reflections on the Classification of Causes of Death’, *Continuity and Change*, 1997, 12, 2, 169–73; George Alter and Ann Carmichael, ‘Classifying the Dead: Toward a History of the Registration of Causes of Death’, *Journal of the History of Medicine and Allied Sciences*, 1999, 54, 2, 114–32.

6 Moriyama I. M., Loy R. M., and Robb-Smith A. H. T., *History of the Statistical Classification of Diseases and Causes of Death* (Hyattsville, National Center for Health Statistics, 2011).

7 Hilde Leikny Sommerseth, ‘What Was Killing Babies in Trondheim?’, manuscript under revision for publication in a special issue for *Historical Life Course Studies*, edited by Angélique Janssens and Alice Reid. See also the article by Leikny Sommerseth and Walhout in this special issue for more details on Norwegian registration practices.

8 Karel Velle, ‘Statistiek en sociale politiek: de medische statistiek in België in de 19de eeuw’, *Belgisch Tijdschrift voor Nieuwste Geschiedenis*, 1985, 1–2, 213–42.

9 For more details, see the article in this special issue by Van den Boomen. See also: F. van Poppel and J. P. van Dijk, ‘The Development of Cause-of-Death Registration in the Netherlands’, *Continuity and Change* 12 (1997) 265–87.

10 Moriyama, *History of the Statistical Classification of Diseases,* 12–13.

11 Ann Hardy, ‘“Death Is the Cure of All Diseases”: Using the General Register Office Cause of Death Statistics for 1837–1920’, *Social History of Medicine*, 1994, *7*, 472–92.

12
https://icd.who.int/en (last accessed 14 March 2022).

13 Jacques Vallin and France Meslé, *Les causes de décès en France de 1925 à 1978* (Paris, INED-PUF, 1988); Judith Wolleswinkel-van den Bosch, *The Epidemiological Transition in the Netherlands* (Rotterdam: Erasmus University, 1998).

14 Josep Bernabeu Mestre *et al.*, ‘El análisis histórico de la mortalidad por causas. Problemas y soluciones’, *Revista de Demografía Histórica*, 2003, 21, 1, 167–93.

15 Thomas Mc Keown, *THE rise of Modern Population* (London: Edward Arnold, 1976).

16 The project is hosted by the Radboud Group for Historical Demography (Radboud University) and led by Angélique Janssens. See the project website: https://www.ru.nl/rich/research-rich/research-groups/radboud-group-historical-demography-family-history/networks/ship/.

17 Angélique Janssens, ‘Constructing SHiP and an International Historical Coding System for Causes of Death,’ *Historical Life Course Studies* 2021, 10 (March): 64–70.

18 These eight studies will be published in a special issue for the online journal *Historical Life Course Studies*, edited by Angélique Janssens and Alice Reid. The following towns and cities are included: Amsterdam, Copenhagen, Hermoupolis, Ipswich, Palma de Mallorca, Rostock, Sundsvall and Trondheim.

19 Angélique Janssens and Elien Van Dongen, ‘A Natural Female Disadvantage? Maternal Mortality and the Role of Nutrition Related Causes of Death in the Netherlands, 1875-1899’, *TSEG - The Low Countries Journal of Social and Economic History,* 2018, 4, 84–115; Isabelle Devos, ‘Te jong om te sterven. De levenskansen van meisjes in België omstreeks 1900’, *Tijdschrift voor Sociale Geschiedenis*, 2000, 26, 2, 55–75.

